# Integrated decoding hematopoiesis and leukemogenesis using single-cell sequencing and its medical implication

**DOI:** 10.1038/s41421-020-00223-4

**Published:** 2021-01-05

**Authors:** Pengfei Qin, Yakun Pang, Wenhong Hou, Ruiqing Fu, Yingchi Zhang, Xuefei Wang, Guofeng Meng, Qifa Liu, Xiaofan Zhu, Ni Hong, Tao Cheng, Wenfei Jin

**Affiliations:** 1 Department of Biology, Southern University of Science and Technology, Shenzhen, Guangdong China; 2 State Key Laboratory of Experimental Hematology & National Clinical Research Center for Blood Diseases, Institute of Hematology & Blood Diseases Hospital, Chinese Academy of Medical Sciences & Peking Union Medical College, Tianjin, China; 3 Center for Stem Cell Medicine & Department of Stem Cell and Regenerative Medicine, Chinese Academy of Medical Sciences & Peking Union Medical College, Tianjin, China; 4 Department of Pediatric Hematology, Institute of Hematology & Blood Diseases Hospital, Chinese Academy of Medical Sciences & Peking Union Medical College, Tianjin, China; 5 Institute of Interdisciplinary Integrative Biomedical Research, Shanghai University of Traditional Chinese Medicine, Shanghai, China; 6 Department of Hematology, Nanfang Hospital, Southern Medical University, Guangzhou, China

**Keywords:** Tumour heterogeneity, Genome-wide analysis of gene expression, Tumour immunology

## Abstract

Single-cell RNA sequencing provides exciting opportunities to unbiasedly study hematopoiesis. However, our understanding of leukemogenesis was limited due to the high individual differences. Integrated analyses of hematopoiesis and leukemogenesis potentially provides new insights. Here we analyzed ~200,000 single-cell transcriptomes of bone marrow mononuclear cells (BMMCs) and its subsets from 23 clinical samples. We constructed a comprehensive cell atlas as hematopoietic reference. We developed counterpart composite index (CCI; available at GitHub: https://github.com/pengfeeei/cci) to search for the healthy counterpart of each leukemia cell subpopulation, by integrating multiple statistics to map leukemia cells onto reference hematopoietic cells. Interestingly, we found leukemia cell subpopulations from each patient had different healthy counterparts. Analysis showed the trajectories of leukemia cell subpopulations were similar to that of their healthy counterparts, indicating that developmental termination of leukemia initiating cells at different phases leads to different leukemia cell subpopulations thus explained the origin of leukemia heterogeneity. CCI further predicts leukemia subtypes, cellular heterogeneity, and cellular stemness of each leukemia patient. Analyses of leukemia patient at diagnosis, refractory, remission and relapse vividly presented dynamics of cell population during leukemia treatment. CCI analyses showed the healthy counterparts of relapsed leukemia cells were closer to the root of hematopoietic tree than that of other leukemia cells, although single-cell transcriptomic genetic variants and haplotype tracing analyses showed the relapsed leukemia cell were derived from an early minor leukemia cell population. In summary, this study developed a unified framework for understanding leukemogenesis with hematopoiesis reference, which provided novel biological and medical implication.

## Introduction

Early studies suggested hematopoiesis occurs through a stepwise process from pluripotent, to multipotent, to oligopotent, to unipotent progenitors and finally to mature blood cells based on dissecting their differentiation potentials ex vivo and in vitro settings^1,2^. However, the hematopoiesis model has to be constantly revised to fit conflicting branches arisen from later studies^3–6^. Single-cell RNA sequencing (scRNA-seq) provides unbiased gene expression profiling of individual cells that is highly complementary to the immunological phenotyping approaches^7^. Recent massively parallel scRNA-seq enabled routine analyses of a large number of single cells for inferring cellular heterogeneity and developmental trajectories^8–12^. In particular, single-cell analysis of hematopoietic stem and progenitor cells (HSPCs) demonstrated hematopoiesis is a continuous process rather than discrete stepwise process^13–18^. However, inconsistency persists among those studies, e.g. Velten et al.^13^ proposed the CLOUD-HSPCs model in which HSPCs directly give rise to distinct lineage-committed populations, while Tusi et al.^15^ proposed a continuously hierarchies model.

The dynamics of gene expression during hematopoiesis has to be precisely regulated, and dysregulation may lead to serious disorders such as leukemia^17,19^. Some leukemia patients have received customized therapy based on genetic variants/mutations they carried, leading to efficient killing of leukemia cells. However, leukemia patients are still under the threat of relapse and drug resistance due to leukemia heterogeneity. Analyses of cancer stem cell in chronic myeloid leukemia identified distinct subpopulations of therapy-resistant stem cells^20–22^. Recent single-cell study on acute myeloid leukemia (AML) revealed primitive leukemia cells aberrantly co-expressed stemness and myeloid priming genes^21^. However, our knowledge about the relationship between leukemia cell subpopulations and progression of leukemia are still limited. Especially, study of different leukemia patients leads to different results, or even conflicting conclusions, potentially due to the individual difference. In this study, we constructed a comprehensive cell atlas of hematopoietic cells, and proposed a hierarchically continuous transition model for hematopoiesis. We developed counterpart composite index (CCI) that integrates multiple statistics to map leukemia cells to reference hematopoietic cells, and proposed a model for the origin of leukemia heterogeneity. The identification of the healthy counterparts of leukemia cells by CCI also could predict leukemia subtype and clinical outcome. Single-cell RNA-seq analysis of a patient at diagnosis, refractory, remission, and relapse vividly demonstrated dynamics of leukemia progression.

## Results

### A comprehensive cell atlas of healthy bone marrow mononuclear cells (BMMCs)

**Fig. 1:**
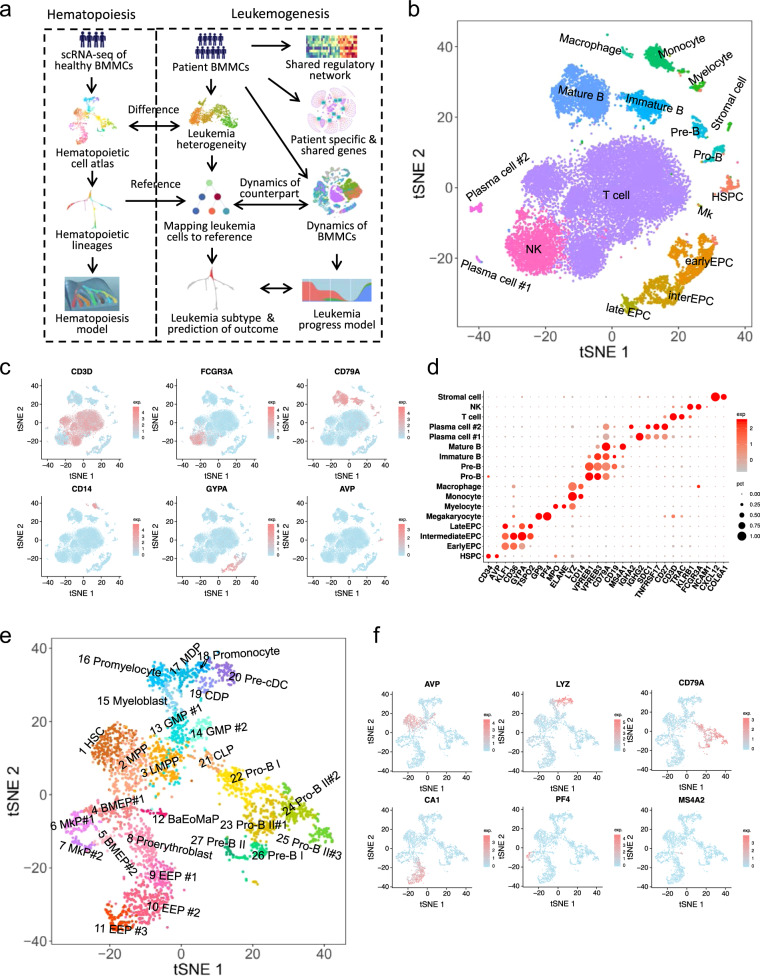
The cell atlas of bone marrow mononuclear cells (BMMCs) and HSPCs (CD34^+^) cells in healthy individuals. **a** The schematic of this study. **b** tSNE projection of BMMCs, colored by inferred cell type. **c** tSNE projection of BMMCs with each cell colored based on their normalized expression of *CD3D*, *FCGR3A*, *CD79A*, *CD14*, *GYPA*, and *AVP*, respectively. **d** Normalized expression level and expression percentage of the cell type-specific genes in 17 cell populations in BMMCs. **e** tSNE projection of HSPCs (CD34^+^) cells, colored by inferred cell type. **f** tSNE projection of HSPCs with each cell colored based on their normalized expression of *AVP*, *LYZ*, *CD79A*, *CA1*, *PF4*, and *MS4A2*.

Bone marrow is the primary tissue for blood cell production, and generates hundreds of billions of blood cells and immune cells per day. In order to gain further biological insights into hematopoiesis and leukemogenesis, we established approaches and pipelines for single-cell analysis of BMMCs and its subsets from 5 healthy samples and 18 leukemia samples (Fig. 1a and Supplementary Fig. S1a, c and Table S1). The 18,751 cells from 4 healthy BMMCs were clustered into distinct cell clusters and visualized by t-Distributed Stochastic Neighbor Embedding (tSNE)^23^ (Fig. 1b). We identified the cell type of each cluster based on their specific highly expressed genes (Fig. 1c, d). The frequencies of major identified cell types in BMMCs were essentially consistent with the expectations (Fig. 1b–d): 1.39% HSPCs (*AVP* and *CD34*), 10.15% erythroid progenitor cells (EPCs) (*GYPA* and *KLF1*), 0.08% megakaryocytes (Mk) (*PF4* and *GP9*), 1.25% myelocytes (*ELANE* and *MPO*), 2.91% monocytes (*LYZ* and *CD14*), 17.67% B cells (*CD79A*), 53.11% T cells (*CD3D*), 11.62% natural killer cells (NK) (*FCGR3A* and *NCAM1*), 0.04% stromal cells (*CXCL12* and *COL6A1*). Furthermore, the frequencies of cell types were highly consistent across different samples (*r*^2^ = 0.96; Supplementary Fig. S1d).

Interestingly, we identified two distinct plasma cell populations both highly expressed *SDC1*, *CD27*, and *TNFRSF17* (Fig. 1b, d). Plasma cell #1 highly expressed *IGHG1*, *IGHG2*, *IGHG3*, *IGHG4* and *IGKC*, while plasma cell #2 highly expressed *IGHA1*, IGHA2 and translation associated genes (Fig. 1d and Supplementary Fig. S1e), indicating their different cellular status and functions. We further identified lots of T cell subsets in BMMCs (Supplementary Fig. S1f). Compared with peripheral blood mononuclear cells (PBMCs)^24^, BMMCs contain much more cells in active proliferation (Supplementary Fig. S1g, h).

### HSPCs form a single-connected entity on tSNE projection

Owing to the limited number of HSPCs in BMMCs (Fig. 1b), CD34^+^ cells, representing HSPCs, were enriched by fluorescence-activated cell sorting (FACS) for investigating hematopoiesis. The HSPCs essentially formed a single-connected entity extending in several directions on tSNE projection and were clustered into 27 clusters for better understanding of the hematopoietic process (Fig. 1e). We inferred the cell type of each cluster by checking the expression of hematopoietic lineage-specific genes (Fig. 1f and Supplementary Fig. S2a), such as HSC (*EMCN*, *THY1*, *MEG3*, *HES1*; cluster 1), megakaryocytic progenitors (MkP) (*PF4*, *GP9*; clusters 6, 7), early erythroid progenitors (EEP) (*APOE*, *CD36*, *CA1*; clusters 8-11), neutrophil, monocyte and DC progenitors (*CSF3R*, *MPO*, and *LYZ*; clusters 13–20), lymphoid progenitors (*CD79A*, *IGHM*, *VPREB1*; clusters 21-27) (Fig. 1f and Supplementary Fig. S2a).

Cluster 12 highly expressed mast cell and basophil-specific genes, including *HDC*, *TPSAB1*, and *MS4A2*, as well as eosinophil-specific gene including *PRG2* (Fig. 1f and Supplementary Fig. S2a), which matched Basophil/Eosinophil/Mast progenitors (Ba/Eo/MaP), a novel cell type has been reported recently^14,24^. We did not detect any cluster with gene expression patterns similar to common myeloid progenitor (CMP) (CD34^+^, CD38^+^, CD123^+^, CD45RA^−^, CD10^−^ and Lin^−^), consistent with recent studies showing that CMP is a heterogeneous mixture of erythroid and myeloid primed progenitors^6,14,25^. Moreover, we observed multiple subpopulations within predefined MkP, EEP, GMP, Pro-B and so on (Fig. 1e). The expression levels of many genes are gradually changing along the three EEP populations, among which the expression levels of *HBA1*, *TFRC*, *GYPA*, *ALAS2*, *PLK1*, and *MKI67* gradually increased as the distance to HSC increased (Supplementary Fig. S2a). Overall, HSPCs contain a substantial higher fraction of cells in active cell cycles and cell states than that of BMMCs (Supplementary Fig. S2b–g). Interestingly, major early stem and progenitor cells (HSC, MPP and LMPP) are in resting phase while major later progenitors are in active proliferation (Supplementary Fig. S2d, e), potentially indicating early progenitors constitute the major cell pool for regulating hematopoiesis while later progenitors are in simple transitional states.

### Continuous hematopoietic lineages with hierarchical structure

**Fig. 2:**
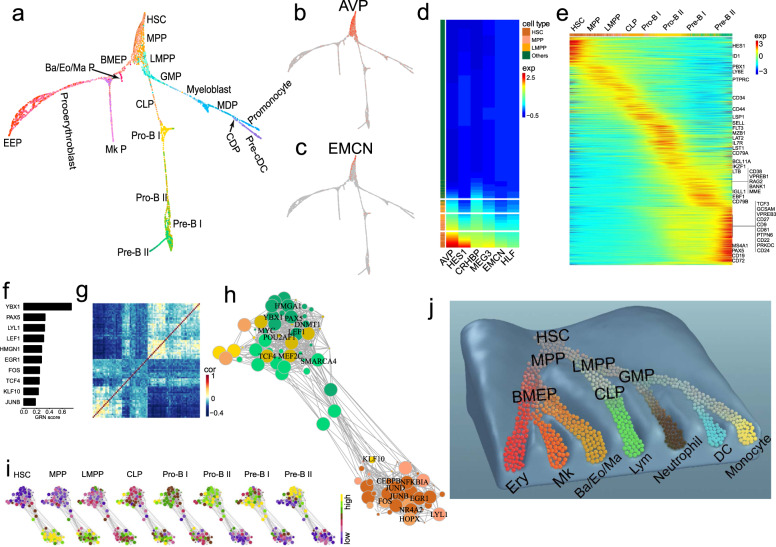
Hematopoietic cell lineages and hierarchically continuous transition model for hematopoiesis. **a** Hematopoietic lineages visualized by SPRING, with cells colored by cell type as in Fig. 1e. **b**, **c** Expressions of *AVP* (b) and *EMCN* (c) are decreasing along hematopoietic lineages. **d** Heatmap of normalized expression level of early hematopoietic markers along lineages. **e** Heatmap of transcriptomic dynamics during lymphopoiesis. **f**–**i** Coordinated TFs and networks underlying lymphoid lineage. The top 10 coordinated TFs (**f**); correlation of the coordinated TFs (**g**); network of coordinated TFs, in which the size of each node represents the magnitude of expression (**h**); dynamics of TFs expression in regulatory networks along lymphoid lineage (**i**), in which each node was colored by average expression level. **j** Hierarchically continuous transition model for hematopoiesis.

We implemented Slingshot^26^ and SPRING^27^ on HSPCs to conduct pseudotime inference. Pseudo-time ordering of HSPCs exhibits a tree-like structure in which HSC forms the root, from which seven lineages gradually emerged with a hierarchical structure (Fig. 2a–c), essentially consistent with the cell lineages based on PCA projection (Supplementary Fig. S3a). The results are consistent with recent reports that hematopoiesis is a continuous process^13–15,28^, while showing different hierarchical structure and lineage relationship compared to previous reports. Clusters 4–5, derived from HSC/MPP, are progenitors of Ba/Eo/Ma lineage, Mk lineage and erythroid (Ery) lineage, were called BMEP, which is consistent with recently identified megakaryocyte–erythroid–mast cell progenitor (MEMP)^29^. This study also showed that neutrophil lineage was derived from GMP while Ba/Eo/Ma lineage was derived from BMEP, different from the classic hematopoietic model in which granulocytes shared a common progenitor^30,31^.

Heatmap analysis showed the expression of early hematopoietic marker genes, such as *AVP*, *HES1*, *CRHBP*, *MEG3*, *EMCN* and *HLF*, are gradually decreasing along pseudotime (Fig. 2d). Although cells were clustered into different populations, their expressions do not show significant changes across the boundaries, further supporting a gradual decrease of stemness along hematopoietic lineage. Attenuations of expression vary greatly from gene to gene, and different cells showed different gene expression patterns (Fig. 2d), indicating each cell holds a unique status during hematopoiesis. Furthermore, by integrating HSPCs and BMMCs together (Supplementary Fig. S3b), we constructed a more comprehensive cell atlas (Supplementary Fig. S3c, d), in which HSPC located in the center while erythrocytes, lymphocytes and monocytes from BMMCs extended at terminal branches (Supplementary Fig. S3e).

### Lineage-coordinated genes, transcription factors (TFs) and networks

We identified thousands of lineage-coordinated genes with expression gradually shifting along the hematopoietic lineages (Fig. 2e and Supplementary Fig. S4a, b). For instance, the expression levels of *CD79A*, *VPREB3* and *PAX5* were increasing along the lymphoid (Lym) lineage (Fig. 2e). The observation that many genes changed continuously along the lineages further supports our presumption of hematopoiesis being a continuous process. We further identified lineage-coordinated TFs of each lineage using gene regulatory networks (GRN) scores^32^. Among the top 10 Lym lineage-coordinated TFs (Fig. 2f), *PAX5*, *LYL1*, *LEF1*, *HMGN1*, *FOS*, and *JUNB* have been reported to play important roles in lymphopoiesis^33^, while *YBX1*, *EGR1*, *TCF4*, and *KLF10* are newly identified. We further identified *SPI1*, *ZEB2*, *CEBPA* and *IRF1*, and *IRF8* in DC lineage; *SPI1*, *CEBPD*, *JUNB*, *CEBPA*, and *KLF2* in neutrophil lineage; *SPI1*, *JUNB*, *CEBPA*, and *FOS* in monocyte lineage; *KLF1*, *GATA1*, *ZEB2*, and *MYC* in Ery lineage; *ZBTB16*, *GATA1*, *ZEB2*, and *KLF2* in Ba/Eo/Ma cell lineage; *FLI1*, *GATA1*, *ZEB2*, and *GFI1B* in Mk lineage (Supplementary Fig. S4c, h).

We constructed lineage-coordinated TF networks based on co-expression network of lineage-coordinated TFs. There are two major subnetworks in Lym lineage-coordinated TF network with strong intra-subnetwork interactions (Fig. 2g, h). The subnetwork usage is gradually shifting from the one highly active in HSC to the one highly active in B-cell progenitor along lymphopoiesis (Fig. 2i). In contrast, there is only one major connective unit in the neutrophil lineage-coordinated TF network, in which TF usage is shifting within the same network during neutrophil genesis (Supplementary Fig. S4d). Overall, we observed two or more subnetworks in Ery, Mk, Lym, and DC lineages, in which active networks were gradually shifting from one subnetwork to another; whereas we only observed one major compact core in neutrophil, monocyte and Ba/Eo/Ma lineages with TF usage shifting in the same network (Supplementary Fig. S4c, h), indicating different models for lineage regulations.

### Hierarchically continuous transition model for hematopoiesis

We propose a hierarchically continuous transition model to explain the hematopoiesis process from HSC to distinct hematopoietic lineages (Fig. 2j). In this model, the hematopoietic system is a dynamic equilibrium system composed of a large number of transitional states/cells, among which contiguous states could mutually convert into each other. Thus, the cell states were only affected by its previous states and compensation effect promotes the HSC transition to vacant slots. The cell fate of a stem cell is not predefined but is gradually determined during cell differentiation. We could consider the classic stepwise model as a specific case of our continuous transition model, in which many transitional states have been missed with FACS-sorted cell population representing some sections of the continuum. The continuous transition model bridges the gap between the classic stepwise models based on FACS sorting and recent continuous models based on single-cell sequencing. Our model provides an essential reference for understanding of leukemia heterogeneity and leukemogenic process.

### Leukemia diversity and shared features among patients

**Fig. 3:**
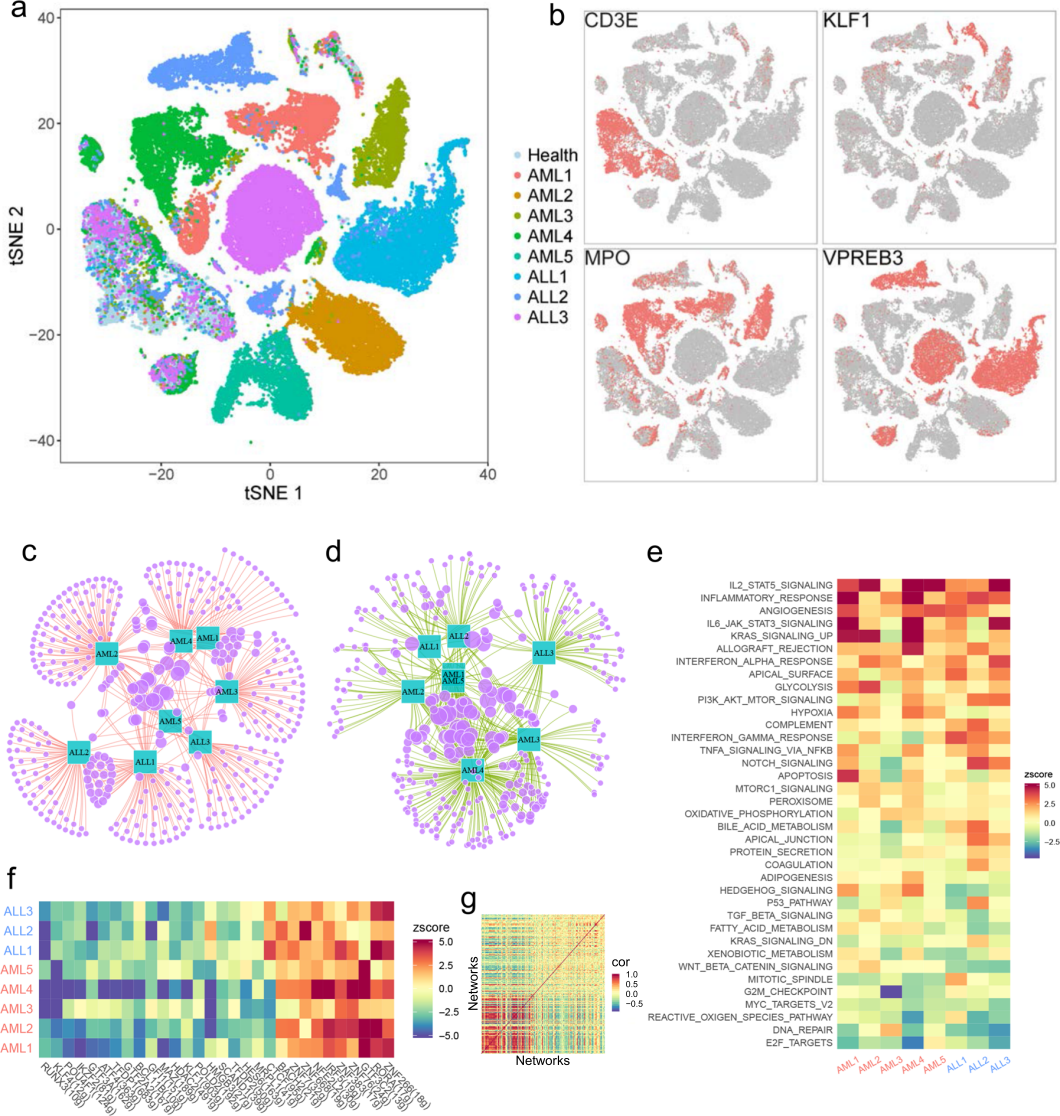
Heterogeneity of leukemia cells and shared features among multiple patients. **a** tSNE projection of BMMCs from 1 healthy individual and 8 leukemia patients, colored by different individuals. **b** tSNE projection of BMMCs from leukemia patients, with each cell colored based on its expression of CD3E, KLF1, MPO, VPREB3, respectively. **c** Majority of upregulated genes among leukemia patients are patient specific. **d** Majority of downregulated genes among leukemia patients are patient specific. **e** Pathway or gene sets commonly upregulated and downregulated among leukemia patients. *Z*-score > 0 means upregulation of pathways while Z-score < 0 means downregulation. **f** Shared upregulated and downregulated TF networks among leukemia patients. *Z*-score > 0 means upregulation of TF networks while *Z*-score < 0 means downregulation. **g** Correlation of TF networks in leukemia patients, in which TF networks were sorted based on their enrichment score decreasingly.

The molecular heterogeneity of the leukemia has significant impact on leukemia classification and treatment^34^. To characterize the heterogeneity of leukemia cells, we investigated the single-cell transcriptomic data of BMMCs from 8 leukemia patients. tSNE projection showed these cells formed two kinds of clusters, either normal cell clusters with cells from both healthy and patient samples, or patient-specific clusters that only comprise of cells from single patient (Fig. 3a). The results indicate leukemia cells are quite different from patient to patient, implying high interpatient diversity. The expression of hematopoietic lineage-specific genes also supports the uniqueness of patient-specific leukemia cells (Fig. 3b). Compared with healthy BMMCs, the majority of the significantly upregulated gene sets or downregulated gene sets are patient specific, with only a few gene sets being shared among multiple patients (Fig. 3c, d).

Although leukemia cells exhibited a high diversity among patients, identification of their shared features may provide important clinical implications for diagnosis and treatment. We noticed *MIR181A1HG*, *ITGA4*, *CD96*, and *TXNP* were upregulated in 5 patients and no genes upregulated in all the 8 patients (Supplementary Fig. S5a). Some gene sets were upregulated or downregulated among all leukemia patients (Fig. 3e). The presence of many shared gene sets while absence of shared genes among all patients indicates patients are genetically specific but share signatures/pathways during cancer progression. For instance, the most significantly upregulated signatures shared by those leukemia patients are IL-2-STAT5 signaling, inflammation response, angiogenesis, IL-6-JAK-STAT3 signaling, KRAS signaling, allograft rejection and hypoxia, which have been reported in various cancers, indicating these signatures played an important role in cancer progression. The most significantly downregulated signatures shared by those leukemia patients are *E2F* targets, DNA repairs, reactive oxygen species pathway, *MYC* targets and G2M checkpoint, indicating reduced cell cycle checkpoint and decreased DNA repair activities play an important role in leukemia. Indeed, the fraction of cells with active cell cycle in leukemia patients is much higher than that in healthy individuals (Supplementary Fig. S5b, e), further indicating higher cell proliferation of leukemia cells.

Compared to healthy BMMCs, we observed the TF networks of *ZNF266*, *RORA*, *GTF3C2*, *ZNF76*, *ZNF383*, *IRF5* and *NFE2L2* being top upregulated in all leukemia patients (Fig. 3f). *RORA* is the key regulator of embryonic development and cellular differentiation, whose upregulation may promote the proliferation of the leukemia cells^35^. *IRF5* promotes inflammation by activating genes producing interferons and cytokines^36,37^. Upregulation of *IRF5* network potentially indicates an increase of inflammation in leukemia patients, although inflammation alone is inefficient to clean up leukemia cells. The top downregulated TF networks sharing among leukemia patients are *RUNX3*, *KLF4*, *POU4F1*, *IKZF2*, *GTF3A*, *ATF4*, *TFDP1*, *GTF2A2*, *BCL11B*, *GFI1*, *MAZ*, *HDAC2* and *KLF1* (Fig. 3f), with majority being hematopoietic lineage specific, indicating that the healthy hematopoietic process was repressed in those leukemia patients. Heatmap analysis showed that upregulated TF networks and repressed TF networks were correlated, although the correlations of repressed TF networks were weaker than that between upregulated TF networks (Fig. 3g).

### Mapping leukemia cells onto reference hematopoietic lineages

**Fig. 4:**
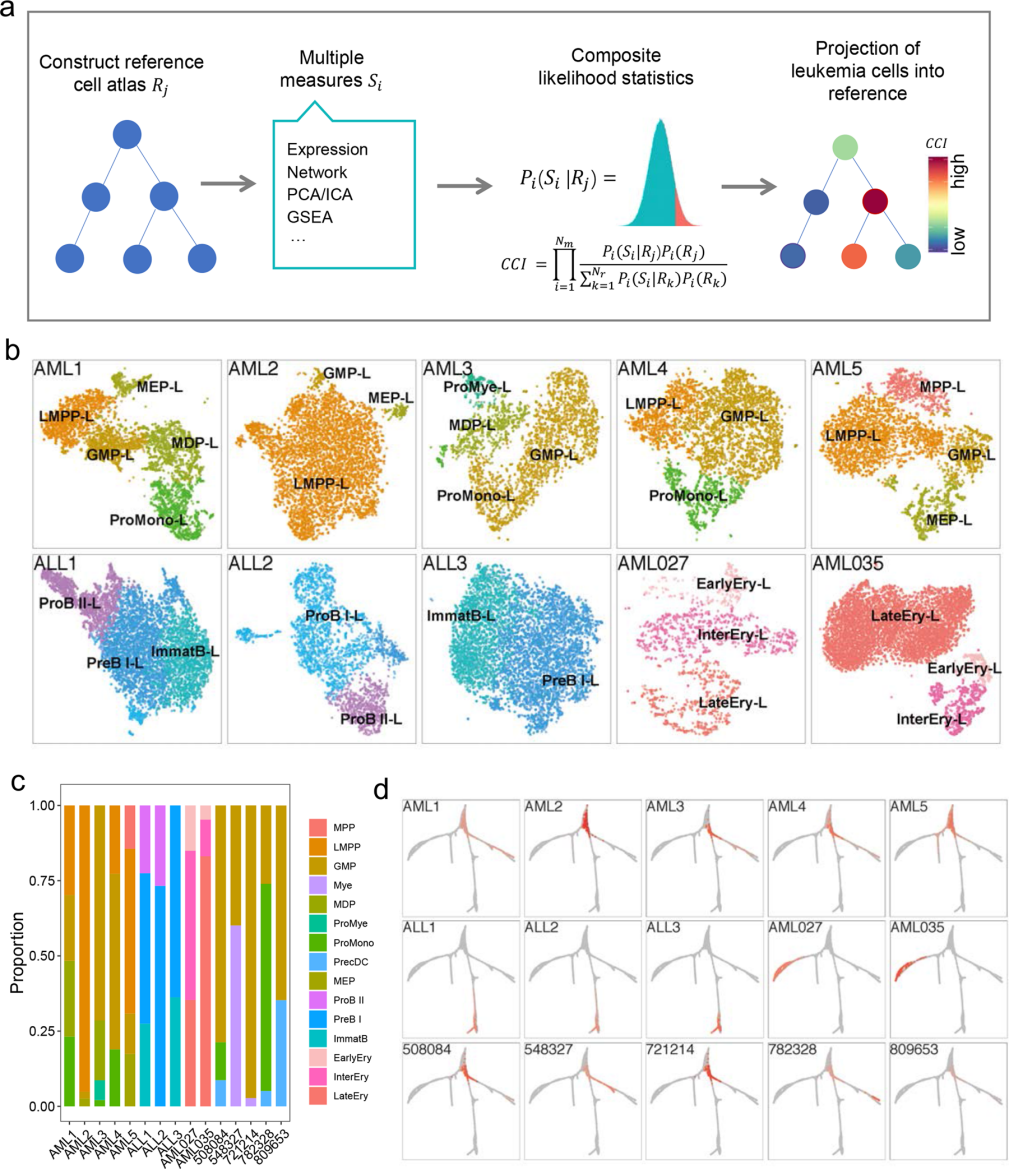
Searching the healthy counterparts of leukemia cells for comprehensive understanding of leukemogenesis. **a** Schematic of CCI for searching healthy counterparts of leukemia cells. **b** The healthy counterpart of each leukemia cell subpopulation in the ten leukemia patients, in which the healthy counterparts of leukemia cells from the sample patient are different. Each leukemia cell subpopulation was named after its counterpart by superscript “-L”. **c** Bar plot of leukemia cell subpopulations and their abundance. **d** Mapping of leukemia cells onto hematopoietic lineages. Gray indicates reference hematopoietic tree while red indicates leukemia cells. The leukemia cells from ALLs were projected into lymphoid lineage, while leukemia cells from AMLs were projected into different non-lymphoid lineages according to their subtypes.

Heterogeneity of the leukemia cells is associated with disease progression and response to chemotherapy^38,39^. Identifying the leukemia cell subpopulations and inferring their origin could facilitate precise diagnosis and treatments. Here, we developed counterpart composite index (CCI) with implementing software (https://github.com/pengfeeei/cci), to search for healthy counterpart of each leukemia cell subpopulation by mapping leukemia cell onto reference healthy hematopoietic lineage (Fig. 4a). CCI integrates multiple statistics, including expression level, co-expression, embedding space of dimension reduction et al., by a composite likelihood statistical framework to improve the statistic power and accuracy (see “Materials and Methods”). Identification of healthy counterparts of leukemia cells not only facilitate our understanding of leukemia progression and leukemia heterogeneity, but also could provide biological insights on the features and functions of the leukemia cell subpopulation via their well-annotated healthy counterparts, thus facilitate prediction of leukemia subtype and clinical outcome.

The substructure of leukemia cells is clearly visible and multiple leukemia cell subpopulations could be observed in each patient (Fig. 4b). The healthy counterparts of leukemia cell subpopulations were different in the same patient and were also different from patient to patient (Fig. 4b, c). The healthy counterparts of leukemia cell in the 12 AML patients include LMPP, BMEP, GMP, MDP, pro-mono, pro-mye, MPP, and Ery progenitors. The leukemia cell subpopulation was named after its healthy counterpart with “-L” suffix. GMP-L, LMPP-L, BMEP-L are the most common leukemia cell subpopulations in AML patients, among which GMP- L showed up in all the 12 AML patients. Interestingly, we found the leukemia cells of two patients (AML027 and AML035) were projected into Ery lineage thus indicated different AML subtypes (Fig. 4b, c). The healthy counterparts of leukemia cell from ALL patients include pro-B II, pre-B I, pre-B II and ImmatB, which belong to lymphoid progenitors. PreB I-L showed up in all the three ALL patients and the heterogeneity of leukemia cells in ALL is weaker than that in AML. Furthermore, the abundances of leukemia cell subpopulations are quite different from patient to patient (Fig. 4c). The accuracy of CCI predictions were validated by cell type-specific genes and also evaluated in another leukemia dataset^21^ (Supplementary Fig. S5f, g).

### Leukemia subtypes, heterogeneity, and stemness based on CCI

Interestingly, mapping of the leukemia cells onto reference hematopoietic lineages showed that leukemia cells from each patient occupy a section of hematopoietic lineage (Fig. 4d), indicating a similar developmental trajectory between leukemia cells and their healthy counterparts. We showed that leukemia cells from different AML patients were mapping into different positions of the hematopoietic tree, such as monocyte lineage (patients: AML1, AML3, AML4, 508084, and 782328), Ery lineage (patients: AML027 and AML035), root (patients: AML2, 721214, and 809653), Mk lineage (patient: AML5) and neutrophil lineage (patient: 548327), indicating different subtypes of AMLs. The AML subtypes inferred by CCI are essentially consist with classic AML subtypes, but with comprehensive information (Supplementary Table S1). The extension and coverage of leukemia cells on hematopoietic tree indicate the heterogeneity of leukemia cells, with long extension or big coverage area indicating high heterogeneity of the leukemia cells, e.g., the AML1, AML4, and AML5 with the highest heterogeneity, while AML2 and 721214 with the lowest heterogeneity due to its shortest extension on hematopoietic tree (Fig. 4d and Supplementary Table S1). Leukemia cells mapping closer to the root of hematopoietic tree means the stronger stemness of leukemia cells, such as AML2, AML5, and 721214. The leukemia cells from ALL patients were projected to lymphoid lineage, which is consistent with the clinical diagnosis while shows different heterogeneity and different stemness (Fig. 4d and Supplementary Table S1).

The patients with leukemia cells projecting into the same hematopoietic lineage showed similar features, while patients with leukemia cells projecting into the different hematopoietic lineage showed much different features. Therefore, projection of leukemia cells into hematopoietic tree is an accurate and unbiased approach for leukemia classification. Furthermore, we found the patient with leukemia cells projecting to the root of the hematopoietic tree were associated with features such as higher fraction of leukemia cells, higher proliferation and higher gene entropy that may lead to worse outcome. Therefore, CCI does not only have the power to infer the subtypes of leukemia, but also provide the heterogeneity and stemness information of the leukemia cells, which could facilitate selection of appreciate treatment.

### Hypothesis about the origin of leukemia heterogeneity

Pseudotime inference of leukemia cells further showed that the trajectories of leukemia cell were similar to that of their healthy counterparts (Supplementary Fig. S5h). In this way, we could assume that leukemia cell subpopulations are not a bunch of independent subpopulations but are a series of lineage-related cells in leukemogenesis. Therefore, we hypothesize the mutant leukemia initial cells or progenitors partially maintain its original developmental trajectory and terminate development at different stages due to loss of different functional genes, leading to a serial of dysfunctional leukemia cell subpopulations, instead of developing into a homozygous population.

### Cell population dynamics of BMMCs in patient ALL3 and the underlying genes

**Fig. 5:**
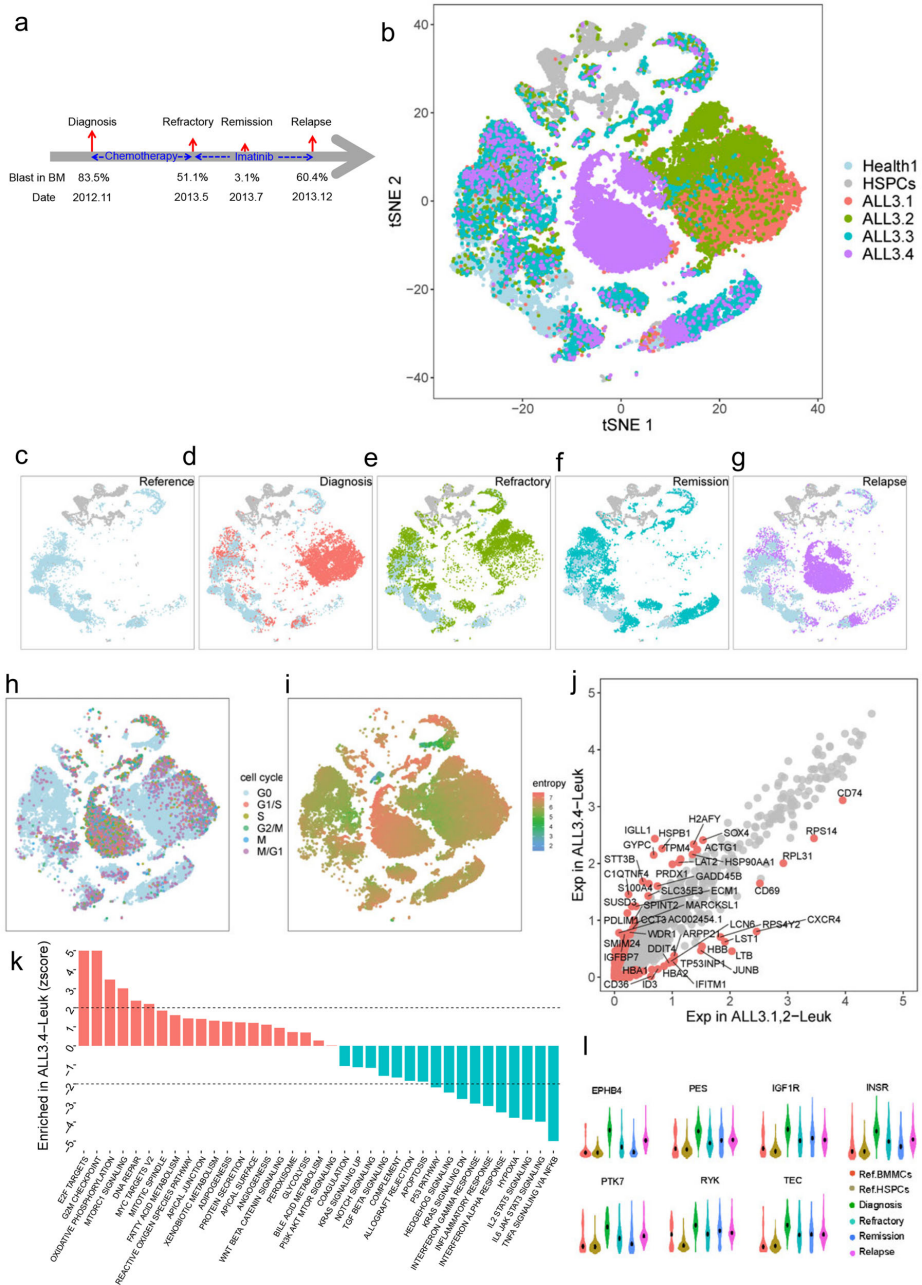
Cell population dynamics of cell populations in patient ALL3 and the underlying genes. **a** Sampling information the patient ALL3 at diagnosis, refractory, remission and relapse. **b** tSNE projection of all BMMCs from this patient and reference cells. **c**–**g** tSNE projection of reference cells and BMMCs at each phase. Reference cells only (**c**), diagnosis (**d**), refractory (**e**), remission (**f**) and relapse (**g**). **h** Cell cycle of patient BMMCs and reference cells. **i** Entropy of patient BMMCs and reference cells. **j** Significantly differential genes between pre-and post-relapse leukemia cells. **k** Significantly enriched pathway and gene sets between pre- and post-relapse leukemia cells. **l** The expression dynamics of some tyrosine kinase (TKs) based on scRNA-seq.

Patient ALL3, a 4-year old boy, was diagnosed with B-precursor ALL in Blood Diseases Hospital, Chinese Academy of Medical Sciences (CAMS)/ Peking Union Medical College (PUMC) in Nov 2012^40^. The patient was refractory to a prolonged chemotherapy, and later achieved remission after Imatinib treatment. The patient relapsed and no longer responded to Imatinib treatment 5 months after remission (Fig. 5a). scRNA-seq data of BMMCs at diagnosis (ALL3.1), refractory (ALL3.2), remission (ALL3.3) and relapse (ALL3.4) were generated, which allowed us to investigate leukemia progression and the underlying mechanisms. By integrating healthy BMMCs and HSPCs as reference, leukemia cells could be easily distinguished from normal cells since leukemia cells are patient specific and normal cells are shared by all samples. Cells from healthy reference formed a notched circle on the tSNE projection (Fig. 5b, c), while leukemia cells formed several distinct clusters at the breach or inside of the notched circle (Fig. 5b). At diagnosis, leukemia cells account for 87.4% of total BMMCs and form a major cell cluster surrounding with some minor cell subpopulations (Fig. 5d). After prolonged chemotherapy, the percentage of leukemia cells in BMMCs slight decrease while the size of some surrounding minor cell subpopulation relatively increased (Fig. 5e). After Imatinib treatment, leukemia cells almost completely disappeared (~1.9%) during remission (Fig. 5f). However, the leukemia cells come back and become dominant (~82.7%) after relapse, with a significant reduction of normal cells (Fig. 5g). Especially, leukemia cells before and after relapse were projected to different coordinates of tSNE projection (Fig. 5b, g), indicating the relapsed leukemia cells are quite different from the leukemia cells in the early stage. The relapsed leukemia cell has the highest fraction of cells in active cell cycle and has the highest entropy among all cell clusters (Fig. 5h, i and Supplementary Fig. S6a), indicating the proliferation and transcriptional complexity of leukemia cells significantly increased after relapse. In summary, these results vividly showed the pronounced dynamics of leukemia cells during clinical treatments and relapse.

According to the above observation, we could conclude that leukemia cells experienced dramatic changes across time. Identifying the differentially expressed genes and pathways between pre- and post-relapse leukemia cells could enhance our understanding of relapse process. We identified 243 significantly upregulated genes and 79 significantly downregulated genes (fold change > 2) after relapse (Fig. 5j). The upregulated genes include *H2AFY*, *IGLL1*, *GYPC*, *HSPB1*, *STT3B*, *C1QTN4*, *SUSD3*, and *PDLIM1* (Fig. 5j), which significantly enriched in *E2F* targeted genes, G2M checkpoints, oxidative phosphorylation, *MTORC1* signaling and so on (Fig. 5k). These gene sets have been reported to promote the cell proliferation in tumor, which is consistent with our observation that relapsed leukemia cells express the highest level of cell cycle genes and show the highest entropy. The 79 downregulated genes include *CXCR4*, *DUSP1*, *JUNB*, *LST1*, *LTB*, *RPS14*, *RPL31*, and *RPS4Y2* (Fig. 5j), which significant enriched in *IL-6-JAK-STAT3* signaling, *TNFA* signaling pathway, *IL-2-STAT5* signaling, interferon alpha response, hypoxia, inflammatory response, interferon gamma response, *KRAS* signaling pathway and hedgehog signaling and so on (Fig. 5k). Imatinib inhibits the enzyme activity of tyrosine kinase that has been shown to play a central role in the pathogenesis of human cancers. We observed the expressions of a lot of tyrosine kinases were changing during the treatment and relapse (Fig. 5l). Therefore, relapsed leukemia cells showed substructure shift and molecular difference with leukemia cells before relapse.

### Leukemia progress model for patient ALL3

**Fig. 6:**
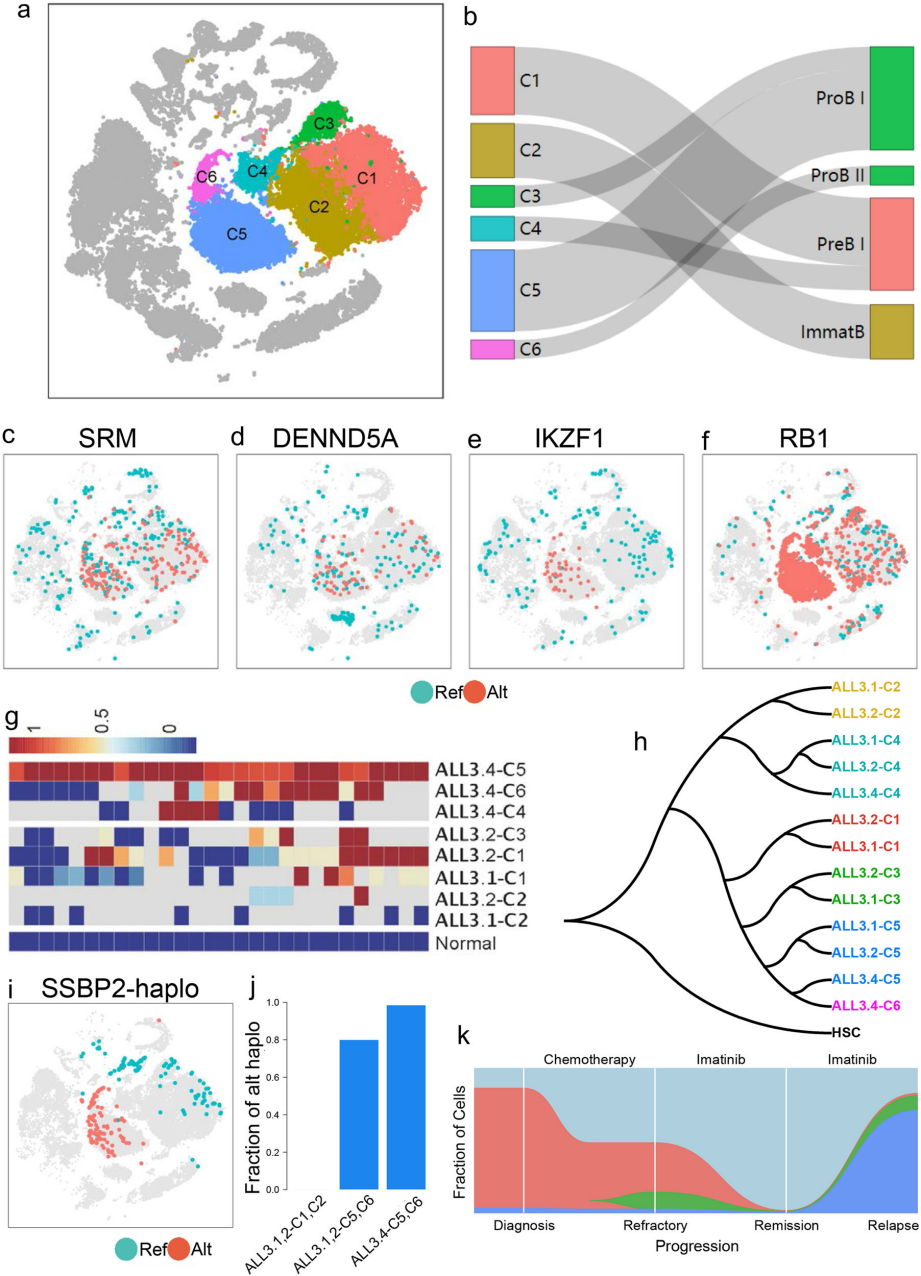
Tracing relapsed leukemia cells and leukemia progression model. **a** Clustering of leukemia cell subpopulations of ALL3. **b** Healthy counterparts of leukemia cell subpopulations, the healthy cell populations are listed by order of lymphoid lineage. **c**, **d** Distributions of leukemia cell-specific high variants on *SRM* (**c**) and *DENND5A* (**d**). **e**, **f** Distribution of relapsed leukemia cell-specific high variants on *IKZF1* (**e**) and *RB1* (**f**). **g** Distribution of genetic variants in different cell subpopulations. **h** Hierarchical tree of leukemia cell subpopulations, in which relapsed leukemia cells and earlier leukemia cells in C5 were clustering together. **i** Distribution of reference haplotype and alterative haplotype of *SSBP2* in BMMCs. **j** Frequencies of relapsed-specific *SSBP2* haplotype (alt haplo) in different leukemia cell subpopulations. **k** Progression model of patient ALL3. Ribbons with different colors present different leukemia subpopulations across the four stages, in which red ribbon stands for major leukemia cells (C1, C2) of diagnosis and refractory stages, blue and purple ribbons stand for relapsed leukemia cells (C5, C6), green is the minor population of leukemia cells from refractory to relapse stage, while light blue is normal cells.

After analyzing the dynamics of BMMCs, we zoomed in leukemia cell subpopulations to provide biological insight on leukemia progress. The total leukemia cells from the 4 time points were classified into 6 subpopulations (Fig. 6a). Using CCI, we found the counterparts of the six leukemia cell subpopulations were B-cell progenitors, namely pro-B I, pro-B II, pre-B, and immature-B (Fig. 6b). The major leukemia subpopulations at diagnosis and refractory (clusters C1 and C2) were pre-B-L and immature-B-L, while the major relapsed leukemia cells (clusters C5 and C6) were pro-B-L (Fig. 6b). Since pro-B is the progenitor of pre-B and pre-B is the progenitor of immature-B in heathy hematopoietic lineage, we could assume that relapsed leukemia cells have increased stemness and stronger differentiation potential than leukemia cells at early stage, which is consistent with our observation that relapsed leukemia cells were in high proliferation states with the highest cell cycle activity and the highest entropy (Fig. 5h, i).

We identified genetic variants from scRNA-seq data at different phases and found increased variants in relapsed leukemia cells (Supplementary Fig. S6b), consistent with the expectation that mutations are progressively accumulated during cancer progress^41^. We identified the leukemia cell or its subpopulation-specific genetic variants by comparing the genetic variants between different cell populations. Among the variants that are significantly different between leukemia cells and normal cells, chr1:11055657;T>C on *SRM* and chr11:9173222;A>C on *DENND5A* are two mutations distributed in all leukemia cell subpopulations and might play an important role in early leukemogenesis (Fig. 6c, d). On the other hand, the mutational alleles on *IKZF1* (chr7:50382590, G>A, p.G158S) and *RB1* (chr13:48409776, T>C) are extremely concentrated in relapsed leukemia cells (Fig. 6e, f), indicating these mutations raised during late phase of leukemia progress. Especially, *IKZF1*^G158S^ is a deleterious mutation associated with leukemia and is a predictor of poor outcome in ALL^42,43^, potentially indicating that *IKZF1*^G158S^ mutation might play an functional role in the relapse of the patient.

We further separate each leukemia cell cluster into multiple subpopulations according to time points of data collection. We found the genetic variants were accumulating during the leukemia progress (Fig. 6g). We further constructed a hierarchical tree based on correlation coefficient of gene expression between pairwise subpopulations, choosing HSCs as the root of the tree. The results showed cell subpopulations from different time points in the same cluster located on the same branch (Fig. 6h). The major relapsed leukemia cells (ALL3.4-C5, ALL3.4-C6) and a minor leukemia cell subpopulation from early phase (ALL3.1-C5 and ALL3.2-C5) are located on the same branch (Fig. 6h), implicating the two subpopulations are similar to each other and potentially shared a common progenitor. In this way, we hypothesize that the relapsed leukemia cells were derived from this minor leukemia subpopulation in early phase (ALL3.1-C5 & ALL3.2-C5) that developed resistance to Imatinib and rapidly expanded to the major subpopulations in relapse. *SSBP2*, a tumor suppressor gene involved in the maintenance of genome stability^44^, contains two haplotypes in the leukemia cells, namely reference haplotype and alternative haplotype. Interestingly, reference haplotypes concentrated in early leukemia cells and normal cells, while alterative haplotypes exclusively enriched in cluster of the major relapse leukemia cells (C5 and C6) (Fig. 6i). We found the fraction of cells with alternative haplotype in early leukemia cells (ALL3.1-C5 and ALL3.2-C5) was nearly as high as that in relapsed leukemia cells (ALL3.4-C5) (Fig. 6j), which strongly supports the notion that relapsed leukemia cells originated from the early minor leukemia cell subpopulation. Furthermore, the genetic variants in leukemia cells subpopulations support that relapsed leukemia cells were derived from minor leukemia cell populations from early phase (Supplementary Fig. S6c, d). Based on these observations, we proposed a model for leukemia progress (Fig. 6k), which vividly showed the dynamics of leukemia cells during diagnosis, refractory, remission and relapse.

## Discussion

Several studies used scRNA-seq to investigate various HSPC recently^13–17^, which greatly increased our knowledge about hematopoiesis. Compared with previous studies, this study focuses on constructing a reference hematopoietic tree for investigating leukemogenesis. We inferred comprehensive hematopoietic process by integrating cluster-specific genes and its niche on hematopoietic tree. We further proposed a hierarchically continuous transition model to fit the hematopoietic development from HSC to 7 hematopoietic lineages (Fig. 2j), in which we vividly showed the transitional cell states, as well as the continuous changes of coordinated genes and TF networks along hematopoietic lineage.

Distinct models have been proposed to explain the origin of leukemia heterogeneity^19,39,45^, However, these studies usually only focus on analyzing relationships among leukemia cell subpopulation thus lost the whole picture of leukemogenesis. Integrating both reference hematopoietic cell and leukemia cell subpopulation has the potential to provide novel insights. However, it is a big challenge to identify the healthy counterparts of each leukemia cell subpopulation based on single signature due to the huge differences between leukemia cell and healthy hematopoietic cells. Consistent results from multiple signatures could potentially generate more reliable results because different measurements provide complementary information about the relationship between cell subpopulation. We developed CCI that integrates multiple measurements for searching the healthy counterpart of each leukemia cell subpopulation in reference hematopoietic cells. Interestingly, we found leukemia cell subpopulations from the same patient had different healthy counterparts. Trajectory analysis of the leukemia cell subpopulations within patient showed linear trajectory (Supplementary Fig. S5h). Mapping leukemia cell subpopulations on the hematopoietic tree showed leukemogenesis almost was exactly the same as truncated hematopoietic development. Therefore, we hypothesize the mutant stem cells partially maintain its original developmental trajectory, while it terminates development at different stages resulting in a serial of different but related leukemia cell subpopulations. Furthermore, identification of the healthy counterparts of leukemia cells not only facilitate our understanding of the leukemia progression and leukemia heterogeneity, but also provide biological insights on the features and functions of the leukemia cell subpopulation via their well-annotated healthy counterparts. Finally, identification of the healthy counterpart of leukemia cell subpopulations have a lot clinical implication such as prediction of leukemia subtype and clinical outcome.

Analyses of clinical data with multiple time points have the potential to provide details about leukemia progressions. Our analyses of patient ALL3 with data at four time points, namely diagnosis, refractory, remission and relapse, vividly showed dynamics of cell population shifting during treatment and relapse. Majority of leukemia cells was killed after Imatinib treatment and the patient achieved remission, However, a minor leukemia cell subpopulation, with the highest similarity to malfunction pro-B, developed Imatinib resistance and later rapid expansion led to relapse. Variants calling from single-cell transcriptomes identified the leukemia-specific mutations and relapsed leukemia cells specific mutations that are potentially associated with leukemia progressions. The relapsed leukemia cells were closer to the root of hematopoietic tree than that of other leukemia cells, thus increased stemness and proliferation. In summary, our study vividly showed that leukemia heterogeneities were associated with cancer progression and therapy outcomes. Application of our single-cell epigenomics approaches on these samples^46,47^ in the future could enhance our understanding of the underlying mechanisms.

In summary, this study developed a unified framework for understanding leukemogenesis, which provided biological insights into leukemia heterogeneity. Furthermore, CCI provided a novel approach for leukemia subtype classification and clinical outcome prediction.

## Materials and methods

### Clinical samples

In total, 23 BMMCs samples and 1 PBMC sample were analyzed in this study, with detail information (Supplementary Table S1). Among them, 14 samples were collected in Blood Diseases Hospital, CAMS/PUMC and Nanfang Hospital, Southern Medical University. This study was approved by IRB of Southern University of Science and Technology (SUSTech). All individuals signed an informed consent form approved by the IRB of the Blood Diseases Hospital, CAMS/PUMC and SUSTech. The other nine BMMCs samples were from other studies^22,48^. The classic diagnosis of leukemia was established according to the criteria of the World Health Organization^49^. Overall, there are 5 healthy samples, 12 AML samples, 6 ALL samples from 3 patients, among which the patient ALL3 were sampled at diagnosis, refractory, remission, and relapse.

### Cell preparation and flow cytometry

BMMCs were isolated from whole bone marrow aspirate by Ficoll density gradient separation (GE Healthcare), resuspended in 90% FBS + 10% DMSO, and cryopreserved in liquid nitrogen. To prepare cells for FACS, frozen BMMCs vials were thawed in a 37 °C water bath for 2 min. Vials were removed once only a tiny ice crystal was left. Thawed BMMCs were washed and resuspended in 1 PBS and 20% FBS. After recovery, FACS sorting was performed on a Becton Dickinson FACSAria II (BD Biosciences, Denmark) to remove the dead cells. BMMCs were stained with pre-conjugated CD34-PE antibody for 15 min on ice. Non-specific binding was blocked by incubation in FACS buffer (Life Technologies). The unbound antibodies were removed using 5 ml wash buffer. The CD34+ cells were sorted out by FACS Aria™ II. The final concentration of thawed cells was 1 x 10^6^ cells per ml.

### Single-cell library preparation and sequencing

The single-cell RNA sequencing libraries of BMMCs and CD34+ cells from healthy individuals and leukemia patients were generated using 10x genomics. In brief, cell suspensions were loaded on a 10x Genomics Chromium Single Cell Instrument (10x Genomics, Pleasanton, CA) to generate single-cell GEMs. Approximately 20,000 cells were loaded per channel. Single-cell RNA-seq libraries were prepared using the Chromium Single Cell 3^’^ Gel Bead and Library Kit (P/N 120237, 120236, 120262, 10x Genomics) following the protocols^48^. Sequencing libraries were loaded at 2.4 pM on an Illumina HiSeq4000 or Illumina NovaSeq 6000 with 2 × 75 paired-end kits.

### Alignment, demultiplexing, unique molecular identifiers (UMI) counting, and normalization

The Single Cell Software Cell Ranger Suite 2 was used to perform reads alignment, barcode demultiplexing, transcripts assemblies and expression counting (https://support.10xgenomics.com). The gene-cell barcode matrix was filtered based on number of genes detected per cell (any cells with less than 500 or more than 4000 genes per cell were filtered out), and percentage of mitochondrial UMI counts (any cells with more than 10% of mitochondrial UMI counts were filtered out). UMI normalization was performed by first dividing UMI counts by the total UMI counts in each cell, followed by multiplication with the median of the total UMI counts across cells. Then, we took the natural log of the UMI counts.

### Dimension reduction and clustering analysis

Dimension reduction was performed by PCA and tSNE^50^, as our previous reports^12,51^. The highly variable genes were inferred based on normalized dispersion following Zhou et al.^51^ The top 30 principal components (PCs) were chosen for tSNE and clustering analysis, according to the explained variances. Single-cell clustering was performed by k-nearest neighbor (KNN) graph and Louvain algorithm. Raw clusters with few differential expressed genes were merged to avoid excessive classification.

### Inferences of hematopoietic lineages and lineage-coordinated genes

Hematopoietic lineages and cell pseudotime of each hematopoietic lineage was inferred by Slingshot^26^, in which PCA was implemented and cell clusters were predefined. The cell clusters inferred by KNN graph were used as input for Slingshot to infer cell trajectories. The constructed hematopoietic lineages was visualized by a force-directed layout in SPRING^27^, in which dimension reduction were generated by DiffusionMap^52^. Differentially expressed genes along the pseudotime were identified using negative binomial tests in Monocle2^11,53^, with the smoothing parameter set to three degrees of freedom. For generating heatmaps of gene expression dynamics, the normalized UMI counts were log transformed and smoothed using Loess regression with the degree of smoothing (span) set to 0.75. Heatmaps for gene expression profile clustering were generated using heatmap function in R. While the other graphics were generated using ggplot2 in R.

### Gene regulatory network (GRN) score

The GRN score^32^ reflect the regulator-target relationships in the context of trajectory progression, which ranks transcriptional regulators based on their correlation with the trajectory, the correlation with their predicted targets, and the extent to which target genes are regulated during the trajectory, which defined as below

$${\mathrm{GRN}}_{i,j} = c_{i,j}m_{t,j}n_j,$$

where GRN_*i,j*_ is the GRN score for regulator *i* along trajectory *j*. *c*_*i,j*_ is the mutual information between transcriptional regulator *i* and trajectory *j*, *m*_*t,j*_ is the average mutual information between predicted target gene *t* and trajectory *j*, *n*_*j*_ is the number of predicted targets regulated along trajectory *j*.

### Lineage-coordinated TF networks

Human transcription factors (TFs) were downloaded from TcoF-DB v2^54^. Lineage-coordinated TF networks were constructed following Fletcher et al.^55^. In short, TFs showing significantly gene expression changing along inferred lineage are lineage-coordinated TFs. The co-expression networks of lineage-coordinated TFs was called lineage-coordinated TF networks. A lineage-coordinated TF was added into the lineage-coordinated TF networks if it correlated with at least 5 other lineage-coordinated TFs (Pearson correlation coefficient > 0.1). TFs correlation heatmaps were generated with NMF R package.

### Entropy analysis

Entropy was used to assess the diversity of single-cell transcriptomes. We applied Shannon Index (SI), which quantifies the level of heterogeneity in potency, to measure the disorder of transcriptomes. Entropy of each single cell was defined as

$${\mathrm{Entropy}} = - \mathop {\sum}\limits_{i = 1}^n {p(x_i){\mathrm{log}}_2p(x_i)},$$

where *p*(*x*_*i*_) represents the probability of gene expression *x* = *x*_*i*_, and *n* is the number of genes.

### Gene sets enrichment analysis (GSEA)

GSEA determines whether a priori defined set of genes shows statistically significant differences between two biological states. The original GSEA was developed for gene-expression assays of bulk data^36,56^, which may lost accuracy when directly implement on scRNA-RNA data. In order to take advantage of thousands of single-cell transcriptomes, we designed an approach as below: (1) Gene expression was averaged from 30 random cells from each cluster due to the high dropout rates of scRNA-seq data. Genes were ranked according to their expression level for each set of cells. (2) Recovery curve was created by walking down the gene list, and steps were increased when we encounter a gene in the gene set. Area under the curve (AUC) was computed as the indicator of enrichment for a certain gene set. Moreover, only AUC of top 5000 ranked genes was considered. (3) To compare the different enrichment of two cell populations for a gene set, we calculate the *Z*-score of AUC in one cell population relative to the AUC distribution in another cell population. Gene sets from Msigdb3.0 (Molecular Signatures Database) were used for analysis by our approach.

### Counterpart composite index (CCI)

It is interesting to systematically compare the developmental trajectories of leukemia cells with that of healthy hematopoietic cells. However, searching the counterpart of a leukemia cell subpopulation in healthy hematopoietic cells is very challenging due to the significant difference between leukemia cells and healthy hematopoietic cells. Based on simulated data, our initial analyses showed that different approaches may produce different results. However, different statistics have much higher probability to generate consistent result when two cell populations have counterpart relationship than these when two cell populations have uncertain relationship. Thus, the composite likelihood of the statistics is the highest when two cell populations have counterpart relationship. Here, we developed an approach, called counterpart composite index (CCI) and was available on GitHub (https://github.com/pengfeeei/cci), to search for healthy counterpart of each leukemia cell subpopulation by integrating multiple statistics to project leukemia cells into healthy hematopoietic cells. CCI integrates Euclidean distance of gene expression, correlation of gene expression, weighted distance in top PCs and difference of gene set enrichment for composite likelihood statistical framework as below:

$${\mathrm{CCI}} = \mathop {\prod }\limits_{j = 1}^{N_{\mathrm{m}}} \frac{{P_i(S_i|R_j)P_i(R_j)}}{{\mathop {\sum }\nolimits_{k = 1}^{N_{\mathrm{r}}} P_i(S_i|R_k)P_i(R_k)}}.$$

In which, *S*_*i*_ is the score of measurement *i*, *R*_*j*_ is the candidate reference cell population *j*, *N*_r_ is the number of reference cell population, *N*_m_ is the number of composite measurements in CCI.

The computational details of each statistic were described as follows:

1) Euclidean distance of gene expression

Considering the prevalent of dropouts and sample size asymmetry, gene expression level was averaged from 30 random cells from each population. The sampling was repeat by ten thousand times for each pairwise populations, from which the median value of expression difference was used as the statistical measurement of two populations. In the meanwhile, pairwise statistical distribution was built from the sampling and computations.

2) Correlation of gene expression

Spearman’s rank correlation coefficient between cell populations was calculated to measure the similarity of gene expression. The sampling and distribution construction were following the same strategy as above.

3) Weighted distance in top PCs

The top principal components, which could represent the distance between cell populations or even single cells, were integrated into CCI. Euclidean distance in embedding space defined by top 30 eigenvectors was weighted by their explained variances.

4) Difference of gene set enrichment

It is well known that cells with similar function have similar biological process and show similar gene set enrichment. Recovery curve of each gene set was constructed and AUC value of top 5000 ranks were used for the indication of cell similarity. Averaged gene expression from 30 random cells of each population were used to calculate the responding AUCs of 50 hallmark gene sets from Msigdb. Median value and statistical distribution were derived from ten thousand of samplings.

Facilitated by the comprehensive cell atlas of BMMCs and HSPCs, we could identify the healthy counterparts of each leukemia cell subpopulation in HSPCs using CCI, which could greatly benefit our understanding of the progression of leukemia.

### Identifying the genetic variants in single-cell transcriptome

There are a few reads in each locus of the single-transcriptomic data; thus, it is very difficult to directly detect the cell-specific variants. In order to reliably detect single-cell expressed specific variants, reads from every single individual were pooled to do variants calling. An in-house script was used to assign each single cell with its associated variants, by checking the variant on indexed reads. To reduce the artifacts and false positives, we used following criteria to filter the cell variants: (1) called in at least 20 cells; (2) at least 5 cells with >2 reads sequenced; (3) variants observed in at least 3 cells. We found that the number of detected transcript mutations in each leukemia patient was much higher than that in each healthy individual, partially due to increased number of expressed genes in leukemia cells.

The single-nucleotide variations (SNVs) identification could be affected by the copy number variations (CNVs) in studied regions. We identified genome wide CNVs using bulk whole-genome sequencing data from the patients, and we used the deletion regions to filter the covered homozygous SNVs, as well as the associated signals. CNVnator (v0.3.2) was used to call CNVs from the whole-genome sequencing data, with a depth of ~30X, of ALL3. The somatic CNVs was then generated by comparing CNVs at different time points to CNVs detected from its saliva sample.

### Haplotype tracing

We observed a sequential of homozygous variants of *SSBP2* from the major relapsed leukemia cell subpopulation ALL3.4-C5, which is the alternative haplotype of *SSBP2*. We analyzed 39 polymorphisms in *SSBP2* to investigate the distribution of the alternative haplotype in different cell populations. Any cell with >5 loci showing the same alleles as that in alterative haplotype (*P* < 0.05) were thought containing the alterative haplotype.

## Supplementary information

Supplementary figures

Supplementary tables

## Data Availability

The raw sequence data reported in this paper have been deposited in the Genome Sequence Archive in BIG Data Center, under accession numbers HRA000084. The code and software of CCI are available on GitHub (https://github.com/pengfeeei/cci).

## Supplementary information

**Supplementary Information** accompanies the paper at (10.1038/s41421-020-00223-4).
