# Supplementary material for: Integrated decoding hematopoiesis and leukemogenesis using single-cell sequencing and its medical implication
Source: Cell Discov. 2021 Jan 5;7:2. doi: 10.1038/s41421-020-00223-4 (PMC7788081; doi:10.1038/s41421-020-00223-4)
Supplement: Supplementary file 1 — Supplementary figures [file 41421_2020_223_MOESM1_ESM.pdf]

Supplementary Fig. S1

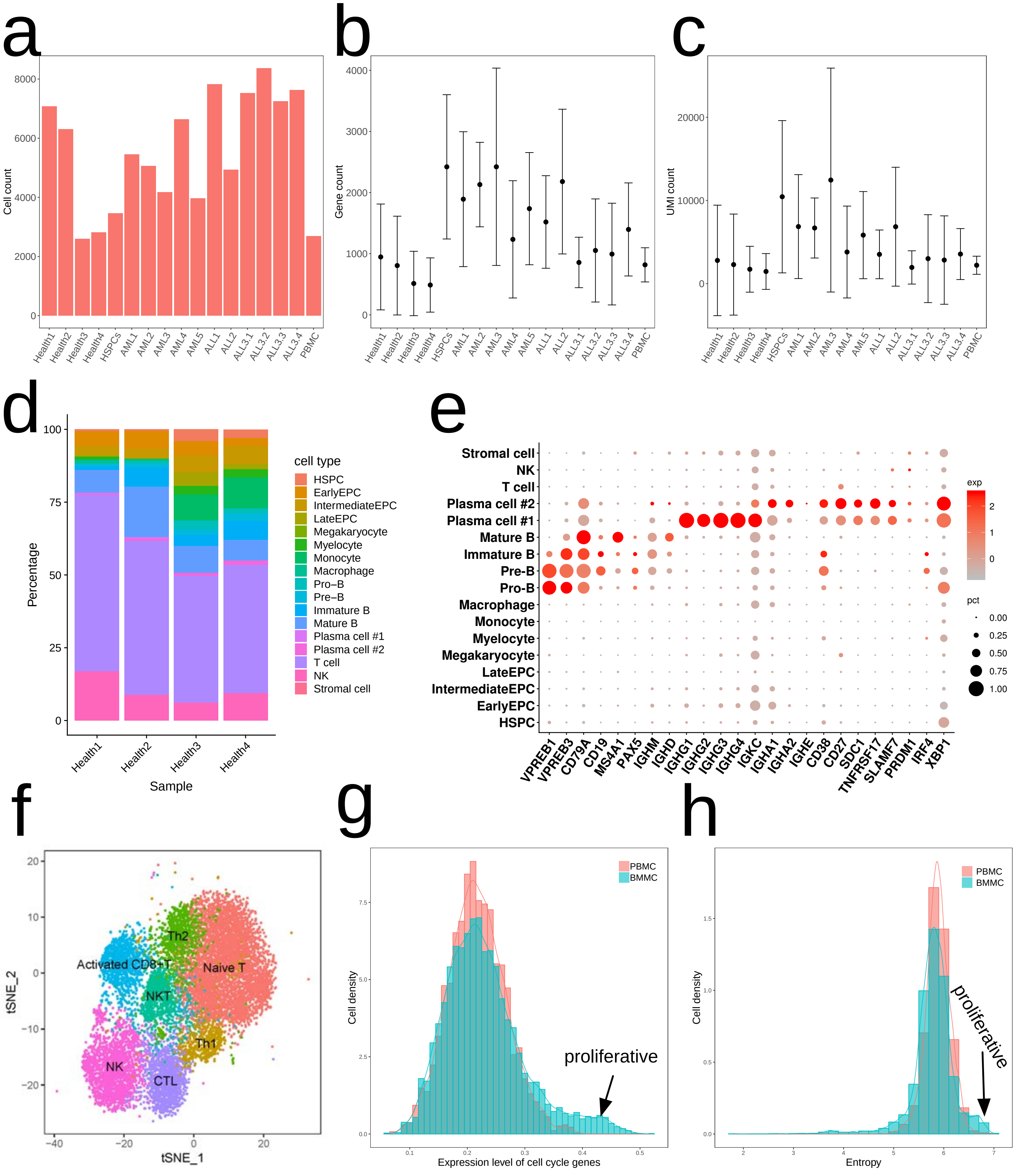

Supplementary Fig. S2

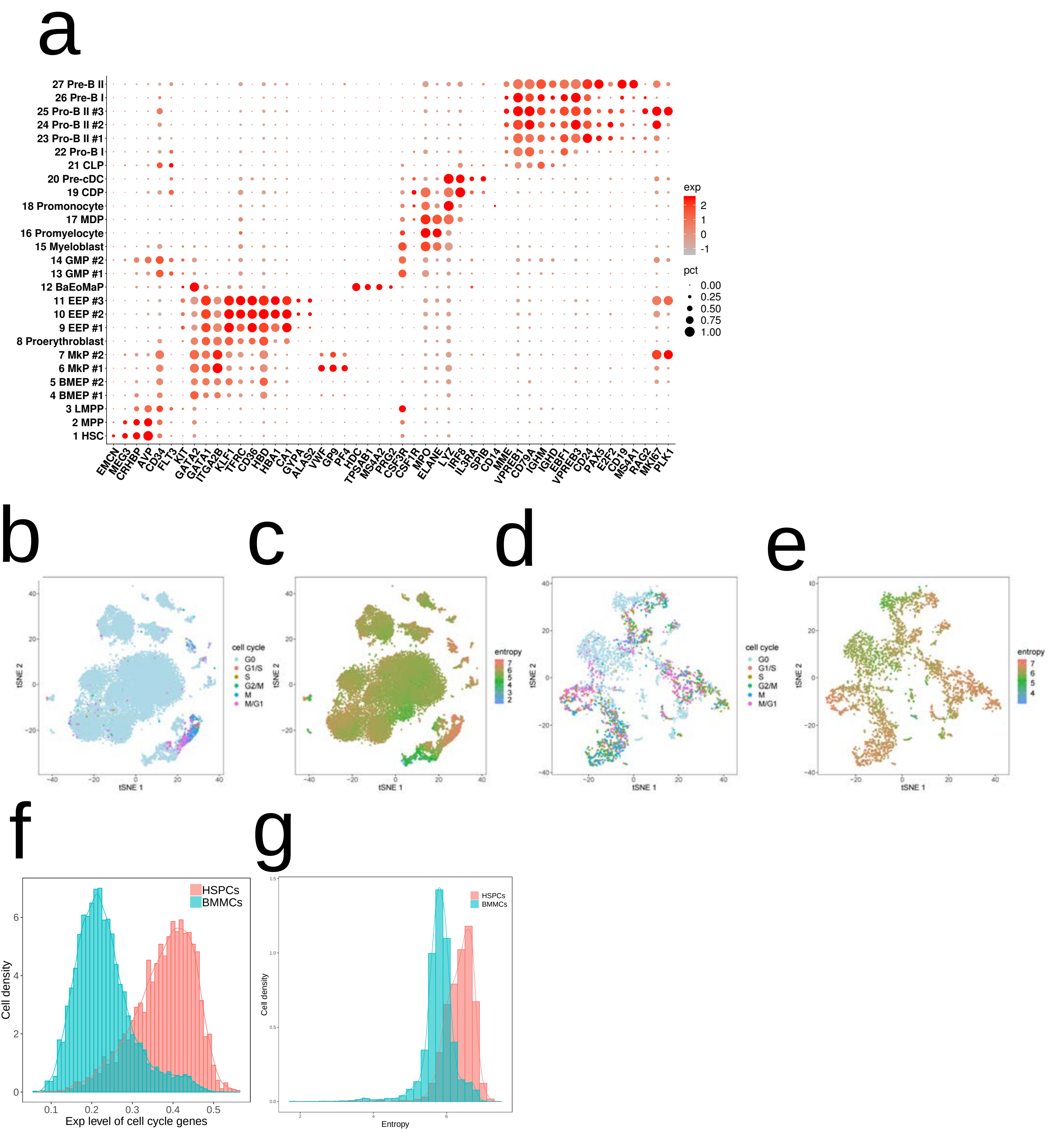

Supplementary Fig. S3

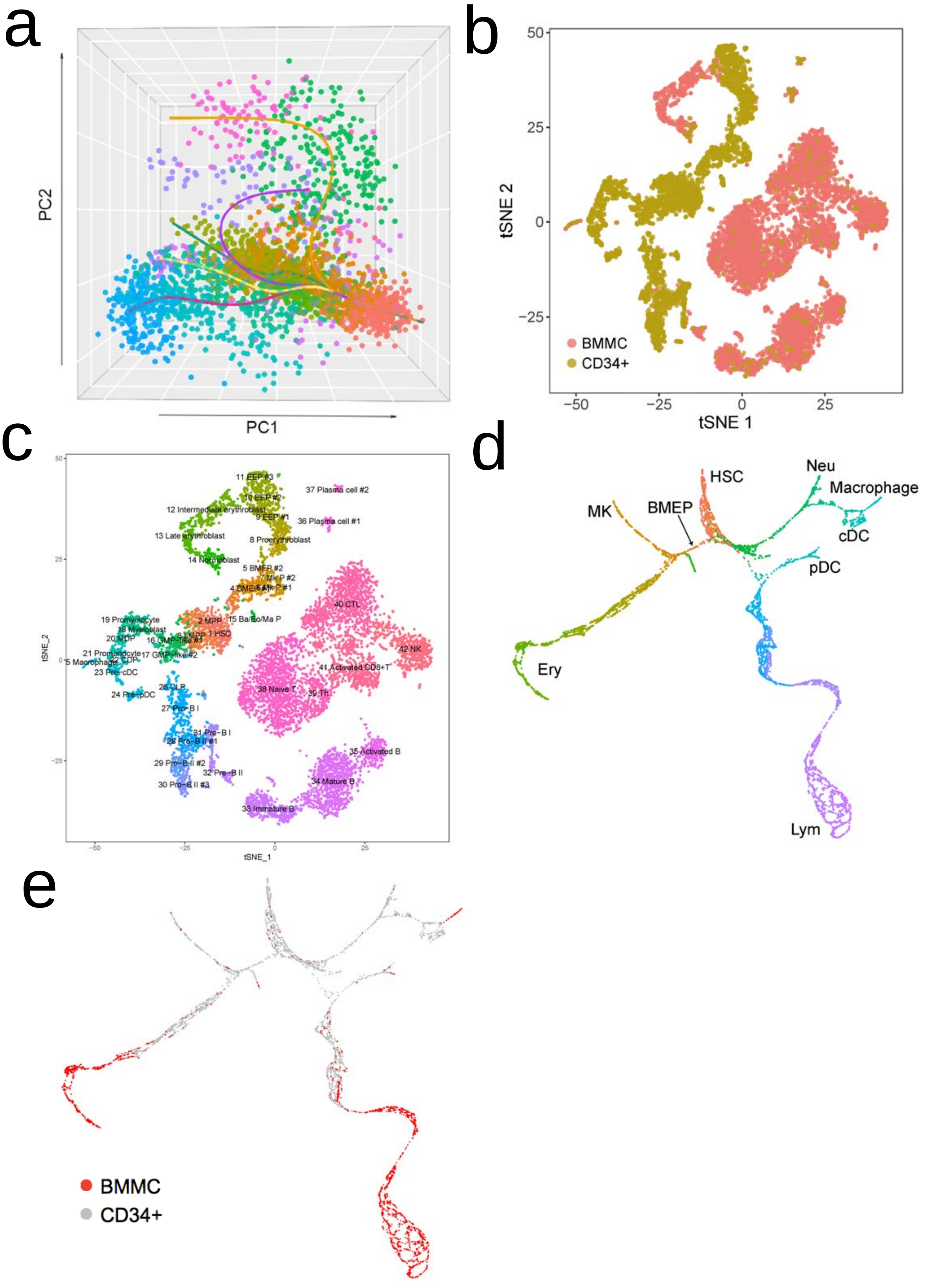

Supplementary Fig. S4

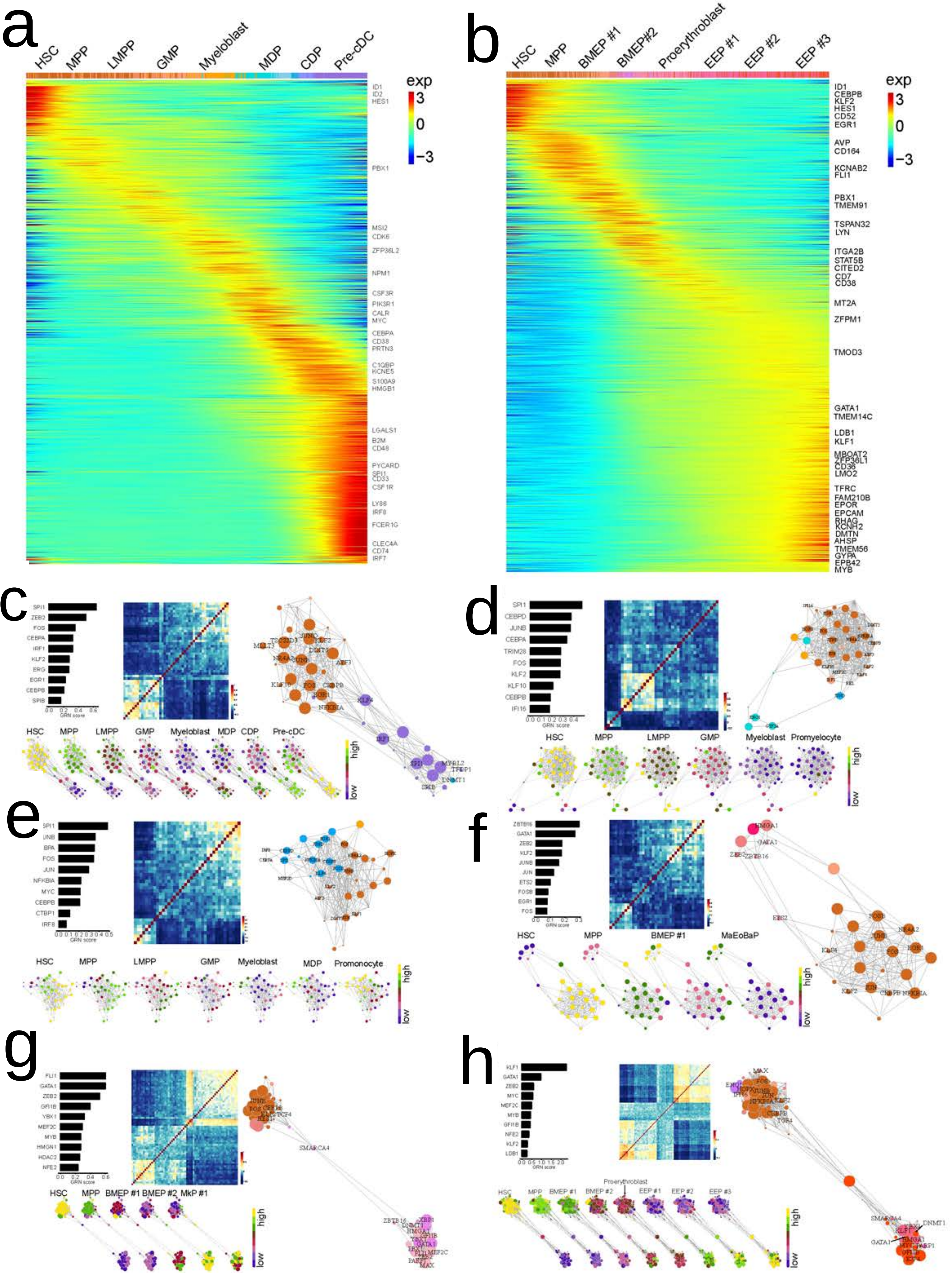

Supplementary Fig. S5

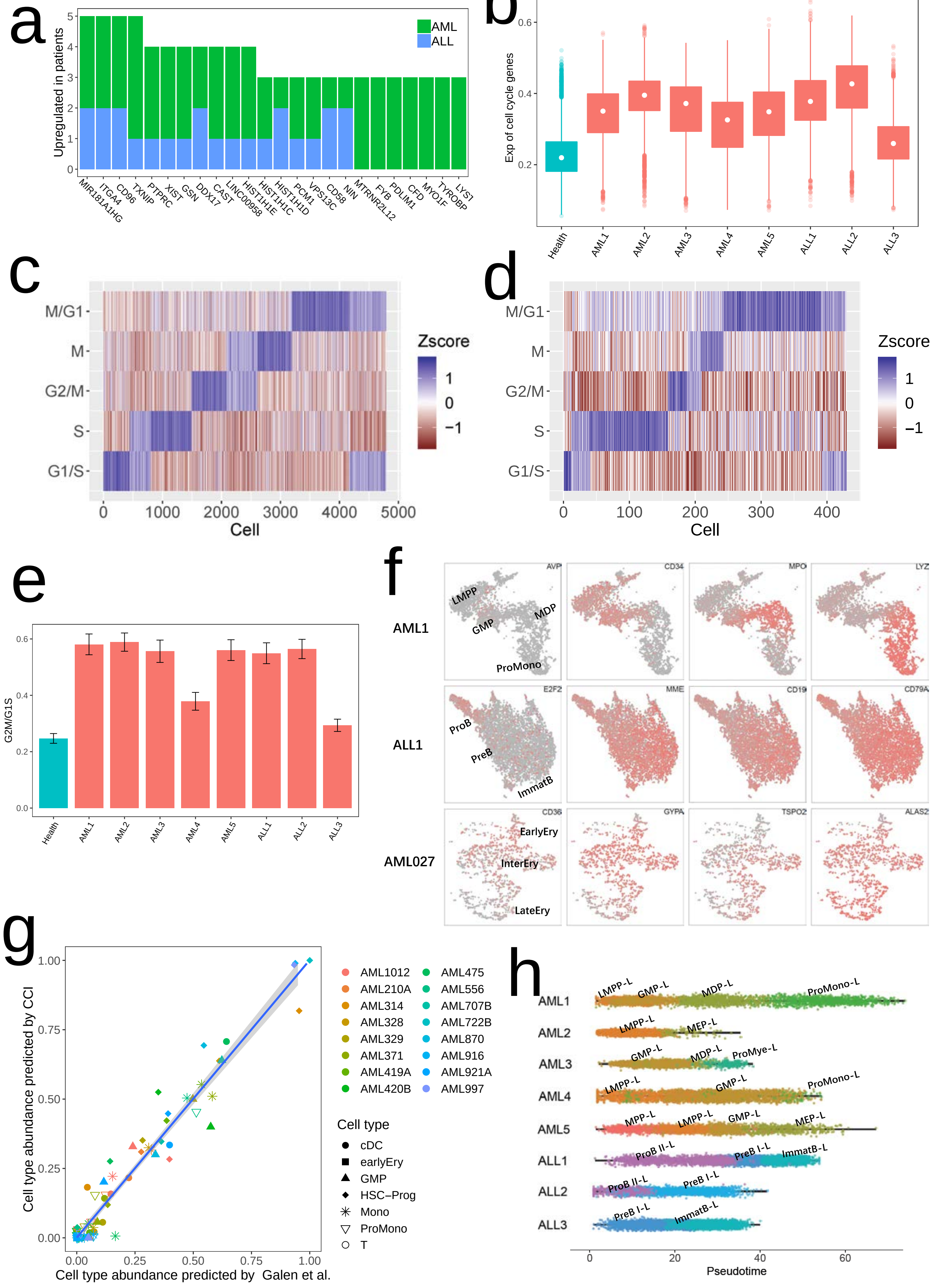

Supplementary Fig. S6

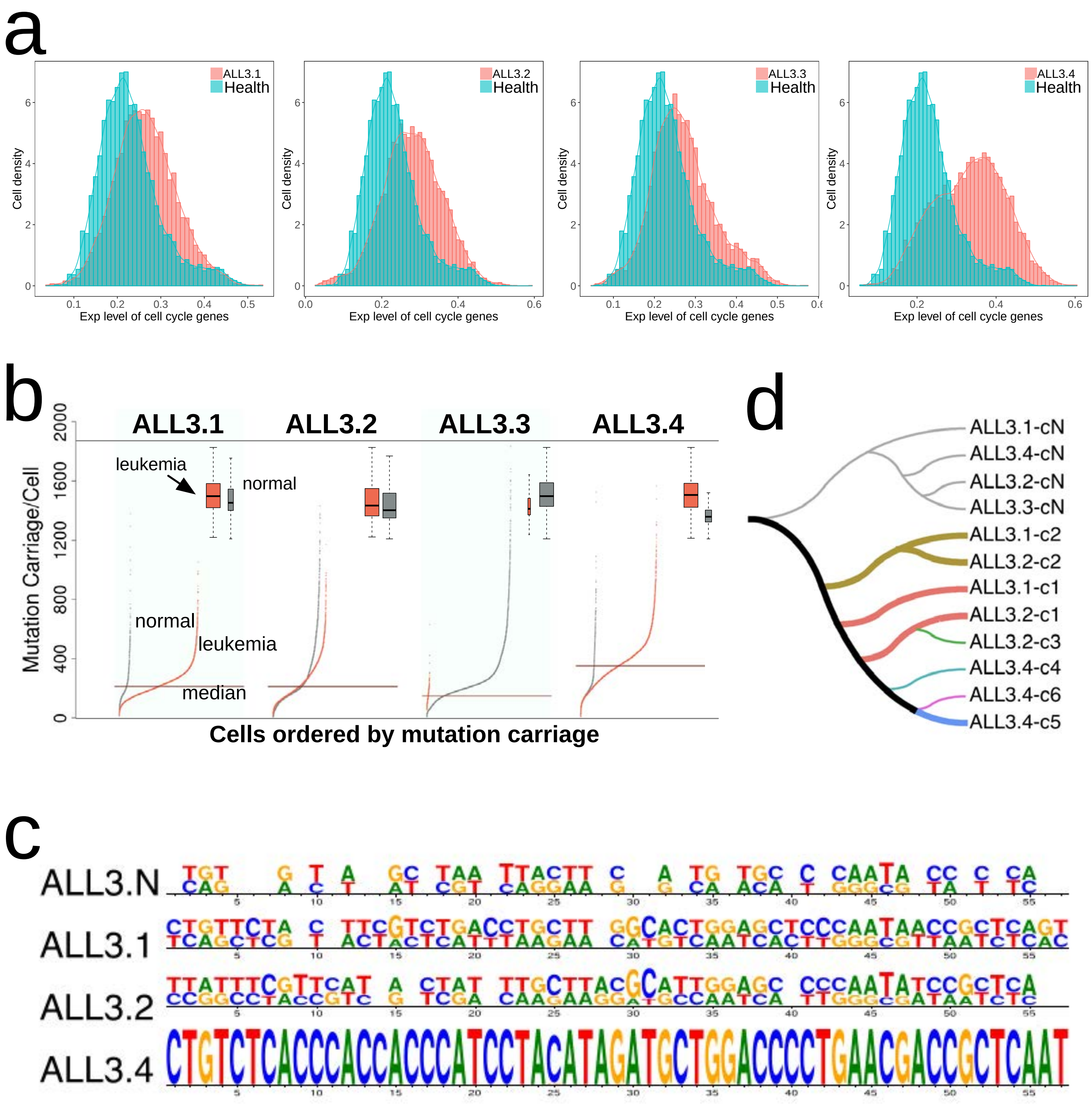

Fig. S1. Basic information of scRNA-seq data and the cell subpopulations in BMMCs.

a-c. Basic data information. Number of cells (a), number of genes (b) and number of UMI (c) after data quality control in all samples.

d. The major Cell types in 4 healthy BMMCs.

e. Differentially expressed genes in two distinct plasma cell populations.

f. T cell subpopulations and NK cells in healthy BMMCs.

g-h. Comparison of cell activity and proliferation in healthy BMMCs and PBMCS revealed by cell cycle (g) and entropy (h) analysis.

Fig. S2. Single cell profiles of healthy BMMCs and HSPCs.

a. Normalized expression level and expression percentage of cell type specific genes in HSPCs subpopulations.

b-c. Cell activity and proliferation in healthy BMMCs revealed by cell cycle (b) and entropy (c) analysis.

d-e. Cell activity and proliferation in healthy HSPCs revealed by cell cycle (d) and entropy (e) analysis.

f-g. Comparison of cell activity and proliferation in healthy BMMCs and HSPCs revealed by cell cycle (f) and entropy (g) analysis.

Fig. S3. Cell atlas and hematopoietic lineages of cells from HSPCs and BMMCs.

a. Trajectory inference of HSPCs by slingshot.

b. tSNE projection of cells from HSPCs and BMMC, colored by HSPCs and BMMCs, respectively.

c. tSNE projection of cells from HSPCs and BMMC, colored by inferred cell types.

d. Hematopoietic lineages were re-inferred by incorporating some BMMC cell populations, which were essentially consistent with that inferred by HSPCs.

e. The HSPCs were located on the core of hematopoietic lineages, while BMMCs (red) extended hematopoietic lineages at branch end.

Fig. S4. Lineage coordinated genes, TFs and TF networks underlying DC lineage, neutrophil lineage, monocyte lineage, Ba/Eo/Ma lineage, Mk lineage and Ery lineage.

a-b. Heatmap of transcriptomic dynamics in DC lineage (a) and Ery lineage (b).

c-h. The top 10 coordinated TFs, correlation heatmap of coordinated TF, connectivity graph of coordinated TFs, dynamics of TFs expression in regulatory networks at different stages with DC lineage (c), neutrophil lineage (d), monocyte lineage (e), Ba/Eo/Ma lineage (f), Mk lineage (g) and Ery lineage (h).

Fig. S5. Features and pseudotime of leukemia cells.

a. Shared high expressed genes among the leukemia patients.

b. Boxplot of expressed cell cycle genes in each cell in healthy BMMCs and leukemia BMMCs.

c-d. Cell cycle pattern of leukemia BMMCs (c) and healthy BMMCs (d).

e. G2M/G1S ratio in healthy and leukemia BMMCs.

f. Expression of marker genes in leukemia cells to validate CCI predictions.

g. Comparison of leukemia cell type predictions between Galen et al. and CCI.

h. Pseudotime of leukemia cells.

Fig. S6. Dynamics of leukemia cells and mutation profile of ALL3 at diagnosis, refractory, remission and relapse.

a. Distribution of expressed cell cycle genes at 4 stages of ALL3.

b. Mutation carriage of each cell at 4 stages of ALL3. Grey dots and box present normal cells of each stage, while red dots and box present leukemia cells. Cells were ordered (x-axis) by their mutation carriage, the median mutation carriage was indicated by the horizontal line.

c. Alleles and haplotypes of *SSBP2* at different cell subpopulations in ALL3.

d. Hierarchical relationship of leukemia cell subpopulation revealed by transcriptional mutations.
